# Prognostic roles of time to positivity of blood cultures in patients with *Escherichia coli* bacteremia

**DOI:** 10.1017/S0950268820000941

**Published:** 2020-05-08

**Authors:** Yufang Chen, Xun Huang, Anhua Wu, Xuan Lin, Pengcheng Zhou, Yao Liu, Yayun Wu, Chenchao Fu, Qingya Dou, Huaye Jiang

**Affiliations:** 1 Department of Hospital Infection Control Center, Xiangya Hospital, Central South University, Changsha, 410008, P. R. China; 2 Department of Hospital Infection Control, Fujian Provincial Hospital, Fuzhou, 350001, P. R. China

**Keywords:** Blood culture, *E. coli* bacteremia, mortality, prognostic roles, time to positivity

## Abstract

The time to positivity (TTP) of blood cultures has been considered a predictor of clinical outcomes for bacteremia. This retrospective study aimed to determine the clinical value of TTP for the prognostic assessment of patients with *Escherichia coli* bacteremia. A total of 167 adult patients with *E.coli* bacteremia identified over a 22-month period in a 3500-bed university teaching hospital in China were studied. The standard cut-off TTP was 11 h in the patient cohort. The septic shock occurred in 27.9% of patients with early TTP (⩽11 h) and in 7.1% of those with a prolonged TTP (>11 h) (*P* = 0.003). The mortality rate was significantly higher for patients in the early than in the late group (17.7% *vs.* 4.0%, *P* < 0.001). Multivariate analysis showed that an early TTP (OR 4.50, 95% CI 1.70–11.93), intensive care unit admission (OR 8.39, 95% CI 2.01–35.14) and neutropenia (OR 4.20, 95% CI 1.55–11.40) were independently associated with septic shock. Likewise, the independent risk factors for mortality of patients were an early TTP (OR 3.80, 95% CI 1.04–12.90), intensive care unit admission (OR 6.45; 95% CI 1.14–36.53), a Pittsburgh bacteremia score ⩾2 (OR 4.34, 95% CI 1.22–15.47) and a Charlson Comorbidity Index ⩾3 (OR 11.29, 95% CI 2.81–45.39). Overall, a TTP for blood cultures within 11 h appears to be associated with worse outcomes for patients with *E.coli* bacteremia.

*Escherichia coli* bacteremia is a common clinical presentation that may at times be transient but on occasion, can lead to septic shock or even death [1]. Established risk factors for mortality include underlying diseases, the severity of systemic inflammatory response syndrome, inadequate antibiotic treatment, nosocomial acquisition, age over 65 years, non-urinary tract source and multi-drug resistant infections [2, 3]. However, such data and other sepsis indicators, specifically elevated levels of C-reactive protein and procalcitonin have limited prognostic precision [4] and some clinical scoring systems may not provide sufficiently timely data or may require intricate calculations not readily appropriate for daily routine use. Therefore, it is very important to identify and utilise a simple and practical indicator to guide clinical treatment. The purpose of this study was to evaluate the prognostic value of the time-to-positive (TTP) interval of blood cultures for patients with *E.coli* bacteremia.

A retrospective observational study was conducted from January 2014 to November 2016 at the Xiangya Hospital Central South University, a 3500-bed comprehensive tertiary hospital located in Changsha, China. Adult inpatients (⩾18 years old) were considered eligible if they had a bloodstream infection with one or more blood culture positive for *E.coli*. Each patient was included only once at the time of the first positive blood culture result, but if multiple blood culture proved positive, the shortest TTP value was recorded. Patients were excluded if they had polymicrobial infections or were treated with antimicrobials prior to blood culture.

Clinical data included age, gender, underlying diseases as per the Charlson Comorbidity Index [5], neutrophil count <1.5 × 10^9^/L, primary site of *E. coli* infection prior to, or coincident with, the onset of bacteremia. The latter was classified as primary in a patient if a specific body site of the acquisition was not evident [6], they had previous surgery or interventional therapy during hospitalisation, had received steroids (prednisolone 10 mg/daily or equivalent dose for a minimum of 2 weeks) and/or other immunosuppressive therapy within 2 months prior to bacteremia. The adequacy of antimicrobial therapy was based on in vitro susceptibility of an isolate and whether antibiotic treatment was started within 24 h after blood cultures were taken. The Pittsburgh bacteremia score [7], presence of septic shock [8] and mortality rates were used to assess clinical outcomes.

At least two sets of blood samples, 20 ml each, were taken from separate venous sites and inoculated into aerobic and anaerobic culture bottles. These were loaded on a BACTEC 9120 automated detection blood culture system (Becton Dickinson, Sparks, MD, USA). All bottles giving a positive signal were examined by Gram staining, subcultured to blood agar medium and incubated for 18–48 h. Isolates were identified to species level and antibiotic susceptibility was determined using the VITEK-2 Compact system（bioMerieux, Marcy L'Etoile, France); susceptibility of the isolates was determined by the MIC, according to the criteria of the CLSI [9]. The TTP for each bottle was defined as the time period from the start of incubation to the alert signal as documented by the monitoring system.

Data analysis was conducted using SPSS for Windows (version 24, SPSS Inc., Chicago, IL, USA). Normality was checked with the D'Agostino's K-squared test. Continuous variables with non-normal distribution were presented as medians with inter-quartile ranges (IQRs) and compared by the Mann–Whitney U-test. Categorical variables, expressed as numbers and percentages, were compared by the Chi-square or Fisher's exact test. The predictive capability of TTP was assessed by receiver-operating characteristic (ROC) and area under the curve (AUC) analysis. The maximum Youden's index [10] was used as a criterion for selecting the optimum cut-off point for TTP. Univariate analysis was performed for associations between risk factors, the incidence of septic shock and in-hospital mortality. Variables with *p* < 0.1 in univariate analysis were entered in multivariate logistic regression models with forward selection. Odds ratio (OR) and corresponding 95% confidence interval (CI) were calculated by standard formulae.

During the study period, 167 patients were included, 72 males and 95 females. The average age was 51.2 (20–88) years and 67 (40.1%) patients were older than 65 years. In total, 26 (15.6%) patients had septic shock and 16 (9.6%) died in the hospital. Relevant clinical characteristics are shown in Table 1.

**Table 1.** Demographical, clinical characteristics of 167 patients with *E.coli* bacteremia

Variable	No. of Patients	% of Patients
Demographics		
Age⩾65 years	67	40.1
Male	72	43.1
Underlying illness		
Malignant tumor	70	41.9
Cardiovascular disease	36	21.6
Diabetes	31	18.6
Chronic liver disease	30	18.0
Chronic kidney disease	11	6.6
Charlson Comorbidity Index⩾3	56	33.5
Neutropenia	36	21.6
Source of infection		
Urinary tract	52	31.1
Intra-abdominal^a^	49	29.3
Others^b^	30	18.0
Unknown	36	21.6
Health-care associated infection	90	53.9
Multidrug-resistant Phenotype	108	64.7
TTP⩽11 h	68	40.7
Recent surgery	31	18.6
Steroid treatment or Chemotherapy	57	34.1
Appropriate empirical antimicrobial therapy	147	88.0
Pittsburgh bacteremia score⩾2	109	65.3
Intensive care unit admission	11	6.6
Septic shock	26	15.6
In-hospital mortality	16	9.6

a Includes intestinal infection, primary or secondary peritonitis, cholecystitis, cholangitis and abdominal abscess.

b Includes respiratory tract, soft tissue, pelvic cavity and intracranial infection.

The median TTP of 167 patients with *E. coli* bacteremia was 12.5 h (range 1.5 to 78.6 h) and the 25th and 75th percentiles were 9.1 h and 18.1 h, respectively. By ROC analysis, the TTP in relation to the occurrence of septic shock showed a significant AUC of 0.71 (95% CI, 0.61–0.80), indicating a moderate predictive capability for this condition (Figure 1). By Youden's index, 11.42 h was found to be the optimal point for TTP and so 11 h was used as the standard cut-off; this value had a sensitivity of 73.1% and a specificity of 65.2% for predicting septic shock. Patients were therefore divided into early (TTP ⩽ 11 h) and late (TTP > 11 h) detection groups.

**Fig. 1.**
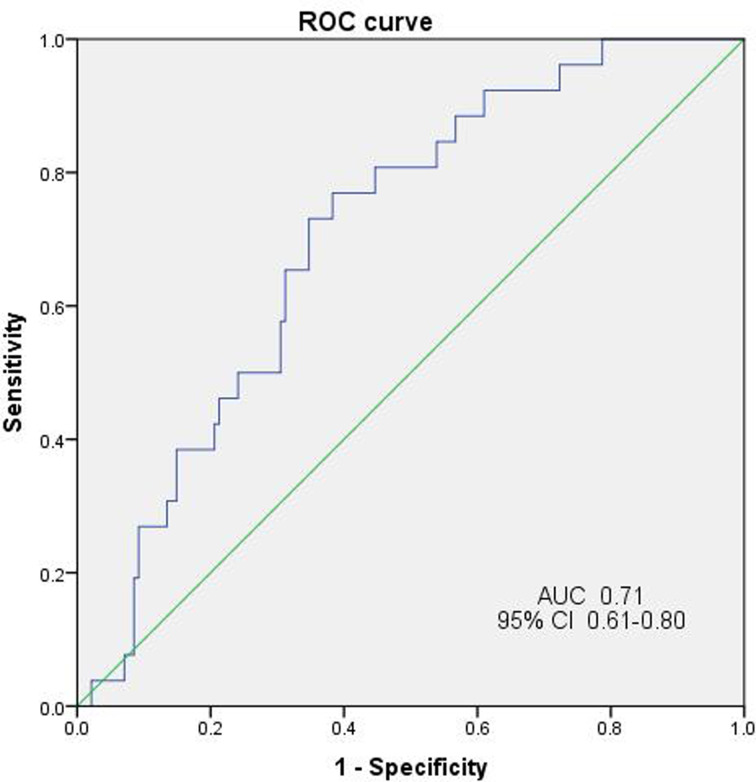
ROC curve of TTP for predicting septic shock.

In terms of clinical characteristics and outcomes of the two TTP groups, patients in the early group had higher Pittsburgh bacteremia scores (⩾2, 45.6% *vs.* 21.2%, *P* = 0.001), a higher incidence of developing septic shock (27.9% *vs.* 7.1%, *P* < 0.001) and significantly higher in-hospital mortality (17.7% *vs.* 4.0%, *P* = 0.003). The median TTP was significantly shorter for patients with septic shock than for those without it (10.30 h, range 3.15–20.30 h *vs.* 16.60 h, range 1.50–78.63 h; *P* = 0.001). Likewise, multivariable logistic regression analysis demonstrated that septic shock was correlated with neutropenia (OR 4.20 95%CI 1.55–11.40, *P* = 0.005), TTP ⩽ 11 h (OR 4.50; 95%CI 1.7–11.93, *P* = 0.03) and admission to intensive care (OR 8.39; 95% 2.01–35.14, *P* = 0.004). Regarding in-hospital mortality, multivariable logistic regression showed that a Charlson Comorbidity Index ⩾3 (OR 11.29; 95% CI 2.81–45.39, *P* = 0.044), TTP ⩽ 11 h (OR 3.80; 95% CI 1.04–13.90, *P* = 0.035), Pittsburgh bacteremia score ⩾2 (OR 4.34; 95% CI 1.22–15.47, *P* = 0.023) and intensive care unit admission (OR 6.45; 95% CI 1.14–36.53, *P* = 0.035) were independent risk factors.

*E. coli* is the most common Gram-negative organism isolated from patients with community-acquired or nosocomial bacteraemia [11,12] which can lead to severe morbidity and mortality. Recently, with the use of automated blood culture systems, the determination of the TTP for patients' blood cultures has been increasingly used as an auxiliary indicator for more precise diagnosis and guide to treatment [13]. Both the bacterial species and their cell numbers can affect the TTP result but as the assay more closely reflects the circulating bacterial load it has the potential to correlate more closely with clinical severity. Our study aimed to investigate whether shorter TTP intervals (early positives) for *E. coli* bacteremia were associated with specific risk factors and poorer clinical outcome of patients when compared with patients characterised by longer TTP intervals (late positives). Analysis of ROC curves is commonly used to judge the accuracy of diagnostic tests and to determine the best cut-off value to distinguish between positive and negative test results. This value is given equal weight to sensitivity and specificity indices and is expressed as the point nearest to the top-left most corner of the ROC curve; also known as the Youden Index. Using this approach, a TTP of 11 h was selected as the standard cut-off and a value ⩽ 11 h was found to have a moderate predictive capability for septic shock, which is similar to the result of an earlier study [14]. However, both the median (12.5 h) and cut-off values of TTP which were indicative of poor prognosis in our patient cohort proved to be substantially longer than those reported by others for *E. coli* bacteremia [15,16]. Such differences might have been due to both patient and strain characteristics as well as the specific methodology of the laboratory assays and clinical diagnostic criteria. Several studies have shown that the shorter the TTP interval, the higher the incidence of septic shock and mortality in bacteremia caused by different bacterial species [16,17] and some have also reported correlations between short TTP intervals and severity of clinical presentation [14–16]. Indeed, our patient cohort with a TTP ⩽ 11 h had higher Pittsburgh bacteremia scores and an increased risk of septic shock as well as greater in-hospital mortality than those with a TTP > 11 h, which was consistent with other studies [14–16]. Our patients with TTP ⩽ 11 h had almost five and fourfold higher risk of septic shock and in-hospital mortality than those with TTP > 11 h, respectively, which supports the view that the chosen cut-off value can be used as a prognostic factor for patients with *E.coli* bacteremia and is therefore of value, in combination with standard diagnostic signs and symptoms, for the assessment of such patients.

We also observed a good correlation between TTP values and clinical outcomes. Notably, neutropenia and intensive care unit admission were independent risk factors for septic shock. Likewise, the Charlson Comorbidity Index, Pittsburgh bacteremia score and intensive care unit admission, which together reflect the severity of underlying diseases and bacteremia, were independently associated with in-hospital mortality in our cohort and was consistent with earlier findings [3].

There are some limitations to our study. Firstly, due to its retrospective nature, our ability to identify clinical variables as significant predictors of mortality was limited. Secondly, TTP is known to be influenced by various factors, such as the sampling time, the blood volume used and the interval from sampling to incubation. Lastly, the sample size of 167 patients is relatively small and the findings need to be validated by prospective studies in a larger series of subjects. In conclusion, we consider that this study has added data which underlines the use of a short TTP is a valuable and clinically relevant index for predicting the occurrence of septic shock, or death, in patients with *E.coli* bacteremia.
